# Supplementary material for: Research Progress of Gliomas in Machine Learning
Source: Cells. 2021 Nov 15;10(11):3169. doi: 10.3390/cells10113169 (PMC8622230; doi:10.3390/cells10113169)
Supplement: Supplementary file 1 [file cells-10-03169-s001.zip › cells-1388631-supplementary.pdf]

Table S1 We retrieved in PubMed Central by using the keywords in this table. (When retrieving, we use not only the abbreviations of the algorithms but also their full names, and only abbreviations are reflected in this table.)

**Machine learning methods**	**Keywords used for retrieval in Pubmed**
support vector machine	((((("glioma"[Title/Abstract] AND "SVM"[Title/Abstract]) NOT "decision tree"[Title/Abstract]) NOT (Naive Bayes [Title/Abstract])) NOT (random forest[Title/Abstract])) NOT (MRMR[Title/Abstract])) NOT (KNN[Title/Abstract])) NOT (neural network [Title/Abstract])
decision tree	((((("glioma"[Title/Abstract] NOT "SVM"[Title/Abstract]) AND "decision tree"[Title/Abstract]) NOT (Naive Bayes [Title/Abstract])) NOT (random forest[Title/Abstract])) NOT (MRMR[Title/Abstract])) NOT (KNN[Title/Abstract])) NOT (neural network [Title/Abstract])
Naive Bayes	((((("glioma"[Title/Abstract] NOT "SVM"[Title/Abstract]) NOT "decision tree"[Title/Abstract]) AND (Naive Bayes [Title/Abstract])) NOT (random forest [Title/Abstract])) NOT (MRMR[Title/Abstract])) NOT (KNN[Title/Abstract])) NOT (neural network [Title/Abstract])
random forest	((((("glioma"[Title/Abstract] NOT "SVM"[Title/Abstract]) NOT "decision tree"[Title/Abstract]) NOT (Naive Bayes [Title/Abstract])) AND (random forest [Title/Abstract])) NOT (MRMR [Title/Abstract])) NOT (KNN[Title/Abstract])) NOT (neural network [Title/Abstract])
MRMR(minimum-redundancy-maximun-relevance)	((((("glioma"[Title/Abstract] NOT "SVM"[Title/Abstract]) NOT "decision tree"[Title/Abstract]) NOT (Naive Bayes [Title/Abstract])) NOT (random forest [Title/Abstract])) AND (MRMR

	[Title/Abstract])) NOT (KNN[Title/Abstract])) NOT (neural network [Title/Abstract])
k nearest neighbors	((((("glioma"[Title/Abstract] NOT "SVM"[Title/Abstract]) NOT "decision tree"[Title/Abstract]) NOT (Naive Bayes [Title/Abstract])) NOT (random forest [Title/Abstract])) NOT (MRMR [Title/Abstract])) AND (KNN[Title/Abstract])) NOT (neural network [Title/Abstract])
neural network	((((("glioma"[Title/Abstract] NOT "SVM"[Title/Abstract]) NOT "decision tree"[Title/Abstract]) NOT (Naive Bayes [Title/Abstract])) NOT (random forest [Title/Abstract])) NOT (MRMR [Title/Abstract])) NOT (KNN[Title/Abstract])) AND (neural network [Title/Abstract])
